# Whole-Genome Sequence of the Novel Temperate *Enterobacter* Bacteriophage Tyrion, Isolated from the Gut of the Formosan Subterranean Termite

**DOI:** 10.1128/genomeA.00839-17

**Published:** 2018-01-04

**Authors:** Chinmay Vijay Tikhe, Chris R. Gissendanner, Claudia Husseneder

**Affiliations:** a Department of Entomology, Louisiana State University Agricultural Center, Baton Rouge, Louisiana, USA; b School of Science, University of Louisiana Monroe, Monroe, Louisiana, USA

## Abstract

We sequenced the genome of the novel *Enterobacter* bacteriophage Tyrion isolated from termite gut. The bacteriophage is temperate in nature and belongs to the *Podoviridae* family. Many predicted bacteriophage genes showed similarity to prophage regions of the genomes of *Enterobacteriaceae* bacteria. This is the second bacteriophage isolated from termite gut.

## GENOME ANNOUNCEMENT

Even though the gut bacterial community of various termite species has been well studied, their bacteriophages remain understudied. We previously isolated and sequenced a novel bacteriophage from termite gut predicted to represent a novel cluster of lytic bacteriophages (1, 2). Here, we report the genome sequence of a novel circularly permuted bacteriophage, Tyrion, infecting *Enterobacter* sp. CT7 (GenBank accession no. KT204538), both isolated from the gut of the Formosan subterranean termite *Coptotermes formosanus*.

Bacteriophage isolation and DNA extraction were carried out as described previously (1). Purified phage DNA was sequenced at Molecular Research LP (Shallowater, TX) using an Illumina MiSeq (2 × 300 bp) platform. The raw reads were checked for quality and adapter contamination using Trim Galore (3) and then assembled into contigs using a SPAdes genome assembler (4). DNA ends were conformed via PCR. Genes were predicted using GeneMark (5) and were manually annotated using the NCBI protein nr database. The family of phage Tyrion was predicted using VIRFAM analysis (6). Electron microscopy was carried out at Socolofsky Microscopy Center at Louisiana State University.

Bacteriophage Tyrion produced small turbid plaques on *Enterobacter* sp. CT7 indicating its temperate nature. Genome sequencing produced a contig with terminal redundancy, and PCR analysis confirmed the DNA ends to be circularly permuted. The genome of phage Tyrion is 41,760 bp, with a G+C content of 51%. At the nucleotide level, segments of the phage Tyrion genome showed similarity to multiple *Escherichia coli*, *Salmonella*, and *Klebsiella* genomes (query coverage 64 to 28%, identity 87 to 76%) and multiple *E. coli* and *Salmonella* bacteriophages (query coverage 63 to 24%, identity 86 to 78%). We were not able to detect any tRNA genes in the genome. The genome of phage Tyrion contained 52 predicted protein-coding genes, of which 51 matched to proteins from prophage-like regions in multiple *Citrobacter*, *Escherichia*, *Salmonella*, and *Enterobacter* strains. Out of 52 genes, 27 genes encoded hypothetical proteins with unknown functions. The bacteriophage genome architecture was similar to that of *Salmonella* phage SPN1S (7) and *Escherichia* phage phiV10 (8).

The genome of Tyrion contained a DNA packaging module comprising a small and a large terminase subunit. The structural module contained a single copy each of the head-tail connecting protein, the major capsid protein, the head closure protein, and an adaptor protein. The lysis cassette was made up of endolysin, holin, and spanin. The genome also harbored an integrase gene and a gene encoding a recombineering protein, confirming its temperate nature. The replication module comprised a primosomal protein and a replication-associated protein. In addition, the genome had a gene encoding an acyltransferase. This acyltransferase has been shown to alter host surface antigens and provide superinfection immunity in *Escherichia* phage phiV10 (8). Electron microscopy and VIRFAM analysis confirmed phage Tyrion to be a member of the *Podoviridae* family.

The wide distribution of bacteriophage Tyrion-like genes in multiple prophages in the *Enterobacteriaceae* family and the potential ability of bacteriophage Tyrion to provide superinfection immunity make it a good candidate for the study of bacteriophage-host interactions (9).

### Accession number(s).

The complete annotated genome sequence of the *Enterobacter* phage Tyrion can be accessed under the GenBank accession no. KX231829.
